# BEHAVIORAL AND SOCIAL IMPACTS ON DAILY VARIABILITY IN HEALTH AND FUNCTIONING IN OLD AND VERY OLD AGE

**DOI:** 10.1093/geroni/igac059.010

**Published:** 2022-12-20

**Authors:** Anna Jori Lücke, Oliver Schilling, Scott Hofer

## Abstract

In recent years, research has shown that people experience substantial variability in domains such as cognition, health, or social interactions from day-to-day or even moment-to-moment. This variability carries relevant information above and beyond an individual’s mean levels of functioning, revealing, for instance, potential risk factors for healthy aging. Thus, aging research increasingly examined such variations in older adults’ daily lives, aiming to further the understanding of aging processes with insights into short- and long-term predictors of daily health and functioning. In this symposium, we introduce research using repeated daily life assessments from older participants to elucidate behavioral and social impacts on variations in working memory performance, pain and self-rated health, as well as social interaction quality. Luo et al. show that diverse daily activities were linked with higher working memory performance one the same day. Regarding long-term prediction, Schilling et al. found that patterns of alcohol consumption across two decades were only weakly predictive of subsequent short-term variability in daily working memory performance. Turning from cognitive functioning to health, Lücke et al. observed bidirectional links of variations in daily sleep quality with variations in daily pain and health perceptions across several days. Finally, Hülür et al. addressed the role of communication technologies for older persons’ social interactions and found that daily social interaction quality differs with the interaction modalities. Scott Hofer will discuss the implications of the presented findings for our understanding of variability in everyday functioning in old age, considering challenges and opportunities for future research in this field.

